# Genotype of metabolic enzymes and the benefit of tamoxifen in postmenopausal breast cancer patients

**DOI:** 10.1186/bcr993

**Published:** 2005-01-28

**Authors:** Pia Wegman, Linda Vainikka, Olle Stål, Bo Nordenskjöld, Lambert Skoog, Lars-Erik Rutqvist, Sten Wingren

**Affiliations:** 1 Department of Biomedicine and Surgery, Division of Cellbiology, Faculty of Health Sciences, Linköping, Sweden; 2 Department of Biomedicine and Surgery, Division of Oncology, Faculty of Health Sciences, Linköping, Sweden; 3 Division of Cytology, Karolinska Hospital, Stockholm, Sweden; 4 Department of Oncology, Huddinge University Hospital, Stockholm, Sweden

**Keywords:** breast cancer, CYP2D6, polymorphism, SULT1A1, tamoxifen

## Abstract

**Background:**

Tamoxifen is widely used as endocrine therapy for oestrogen-receptor-positive breast cancer. However, many of these patients experience recurrence despite tamoxifen therapy by incompletely understood mechanisms. In the present report we propose that tamoxifen resistance may be due to differences in activity of metabolic enzymes as a result of genetic polymorphism. Cytochrome P450 2D6 (*CYP2D6*) and sulfotransferase 1A1 (*SULT1A1*) are polymorphic and are involved in the metabolism of tamoxifen. The CYP2D6*4 and SULT1A1*2 genotypes result in decreased enzyme activity. We therefore investigated the genotypes of *CYP2D6 *and *SULT1A1 *in 226 breast cancer patients participating in a trial of adjuvant tamoxifen treatment in order to validate the benefit from the therapy.

**Methods:**

The patients were genotyped using PCR followed by cleavage with restriction enzymes.

**Results:**

Carriers of the CYP2D6*4 allele demonstrated a decreased risk of recurrence when treated with tamoxifen (relative risk = 0.28, 95% confidence interval = 0.11–0.74, *P *= 0.0089). A similar pattern was seen among the SULT1A1*1 homozygotes (relative risk = 0.48, 95% confidence interval = 0.21–1.12, *P *= 0.074). The combination of CYP2D6*4 and/or SULT1A1*1/*1 genotypes comprised 60% of the patients and showed a 62% decreased risk of distant recurrence with tamoxifen (relative risk = 0.38, 95% confidence interval = 0.19–0.74, *P *= 0.0041).

**Conclusion:**

The present study suggests that genotype of metabolic enzymes might be useful as a guide for adjuvant endocrine treatment of postmenopausal breast cancer patients. However, results are in contradiction to prior hypotheses and the present sample size is relatively small. Findings therefore need to be confirmed in a larger cohort.

## Introduction

The majority of breast tumours express oestrogen receptors (ERs). Several studies have shown that 5 years of tamoxifen therapy in breast cancer patients with receptor-positive tumours reduces the risk of recurrence and mortality [1]. However, about 30% of patients acquire tamoxifen resistance and relapse in the disease [1]. Several possible mechanisms for this have been suggested [2-4].

Tamoxifen and its metabolites compete with endogenous oestrogen for the ligand-binding domain of the ER. The complex formation between tamoxifen, or its active metabolites, and the ER inhibits recruitment of co-activator complexes necessary for transcription of oestrogen-responsive genes [5]. The biotransformation of tamoxifen is mediated by cytochrome P450 enzymes mainly through demethylation and hydroxylation to form several primary metabolites, principally 4-OH-tamoxifen, α-OH-tamoxifen, *N*-desmethyl-tamoxifen, and 4-OH-*N*-desmethyl-tamoxifen. 4-OH-tamoxifen is considered to be a more potent anti-oestrogen than the mother substance and is capable of binding the ER with greater affinity [6,7]. From experimental studies it has been shown that the transformation of tamoxifen into 4-OH-tamoxifen is mainly catalysed by the liver enzyme CYP2D6 [8,9]. A further step in the metabolism of tamoxifen is sulfate conjugation, catalysed by members of the sulfotransferase family, which generally increase the solubility and facilitate excretion of the drug. Sulfotransferase 1A1 (SULT1A1) is a major form of phenol sulfotransferase in the adult human liver, and it has been shown to be the primary sulfotransferase responsible for the sulfation of 4-OH-tamoxifen [10,11].

Polymorphisms affecting the enzyme activity have been found in both cytochrome P450 2D6 (*CYP2D6*) and *SULT1A1 *[12,13]. Among Caucasians the most frequent inactivating polymorphism in *CYP2D6 *is the CYP2D6*4 allele, which generates a G → A transition at nucleotide 1934 leading to a disruption of the reading frame and to a truncated non-functional gene product [14]. The most common polymorphism in the *SULT1A1 *gene is a G → A transition at nucleotide 638, resulting in an arginine to histidine substitution at the conserved amino acid 213. This allele, SULT1A1*2, is correlated with diminished capacity to sulfate SULT1A1 substrates [15]. The aim of the present study was to investigate the genotypes of *CYP2D6 *and *SULT1A1 *in breast cancer patients with and without tamoxifen treatment in order to validate the relation between the genotype and the benefit from tamoxifen therapy.

## Materials and methods

### Patients

The Stockholm Breast Cancer Group started a trial in 1976 to compare postoperative radiotherapy with adjuvant chemotherapy [16]. Both premenopausal patients and postmenopausal patients (age ≤ 70 years) with a unilateral, operable breast cancer were included. The patients were required to have either histological verified lymph node metastases or a tumour diameter exceeding 30 mm. All patients were treated with a modified radical mastectomy. Using a 2 × 2 factorial study design, the postmenopausal patients were then randomised to a comparison of adjuvant tamoxifen treatment or no endocrine treatment in a total of four treatment groups: adjuvant chemotherapy, adjuvant chemotherapy plus tamoxifen, radiotherapy, and radiotherapy plus tamoxifen. Tamoxifen was given postoperatively at a dose of 40 mg daily for 2 years and was initiated within 4–6 weeks of surgery. The mean follow-up time was 10.7 years (range, 0.24–18.6 years). Of the 679 postmenopausal breast cancer patients included in the trial, fresh frozen tumour tissues of 226 patients were available for the current investigation, of whom 112 had received tamoxifen therapy. The number of distant recurrences was 64 in the tamoxifen-treated group and 84 in the group not receiving tamoxifen. Furthermore, the fractions of lymph-node-positive and ER-positive tumours were 88/89 and 71/70, respectively, and the percentage of large tumours (>20 mm) was 57/61 in the initial study and the current study.

### Polymerase chain reaction

DNA was isolated from fresh-frozen tumour tissues using phenol, phenol/chloroform (1:1), and chloroform, was precipitated with ethanol and was re-dissolved in sterile water. The *CYP2D6 *and *SULT1A1 *genes were amplified with PCR in separate reactions using 30 ng DNA and 60 ng DNA, respectively. The primer sequences used in the PCR of *CYP2D6 *and *SULT1A1 *were adopted from Hanioka and colleagues [14] and Coughtrie and colleagues [17]. The following PCR reagents were added to a reaction volume of 20 μl: 2 mM MgCl_2_, 0.2 mM dNTPs, 0.5 units *Taq *DNA polymerase, and 1 μM each of forward and reverse primer in 1 × PCR buffer. The amplifications were carried out in a PTC-200 Peltier Thermal Cycler DNA Engine (MJ Research™ Inc, Waltham, MA, USA). An initial denaturation at 94°C for 3 min was followed by 40–43 cycles of 30 s at 94°C, 30 s of annealing at 63°C, and 40 s for extension at 72°C. An extension period of 5 min followed the final cycle.

### Restriction fragment length polymorphism

The *CYP2D6 *and *SULT1A1 *polymorphisms were detected using restriction enzymes. The *Mva*I enzyme distinguishes between the CYP2D6*4 allele and other *CYP2D6 *alleles. The polymorphic allele CYP2D6*4 lacks the restriction site, and is thereby retained as one fragment. Alleles harbouring the *Mva*I restriction site generate two fragments and are classified as CYP2D6*1. SULT1A1*1 (wild-type allele) has a restriction site recognised by the *Hae*II enzyme, while the polymorphic SULT1A1*2 lacks this site.

Ten units of *Mva*I (Fermentas, Stockholm, Sweden) and 1.5 μl R^+ ^Buffer (Fermentas) were added to each tube of *CYP2D6 *PCR products and were incubated at 37°C for 2.5 hours. The *SULT1A1 *PCR products were incubated with 5 units of the restriction enzyme *Hae*II (New England BioLabs, Beverly, MA, USA) in a 20 μl reaction mixture containing 1 × NE (50 mM potassium acetate, 20 mM Tris-acetate, 10 mM magnesium acetate, 1 mM dithiothreitol, pH 7.9) buffer (New England Bioloabs), supplemented with 100 μg/ml BSA. After digestion, fragments were resolved by electrophoresis on a 3% (w/v) agarose gel containing 1 × TBE (89 mM Tris, 89 mM Boric acid, 2 mM EDTA, pH 8.4) buffer and ethidium bromide (0.5 μg/μl). A 100-molecule weigh ladder was used as the base pair marker. The gel was finally processed in a UV detector (Spectromics Corporation, New York, USA). To confirm the reliability of the restriction fragment length polymorphism method, a number of randomly selected samples were DNA sequenced. No differences in genotype were obtained between the methods.

### Statistical analyses

Statistical analyses were performed with the Statistica 6.0 software program (Statsoft Inc., Tulsa, OK, USA). We compared distant recurrence-free survival by genotype and by endocrine treatment with the log-rank test. The relative risk (RR) of distant recurrences among ER-positive patients treated with and without tamoxifen was assessed using Cox proportional hazard regression, and adjustments for age, tumour size, and lymph node status were performed.

## Results

Information on tumour size, nodal involvement, ER status and tamoxifen therapy of 226 patients is presented in Table 1. The patients were genotyped according to the CYP2D6*4 and the SULT1A1*1 or SULT1A1*2 alleles. There were no significant differences in tumour characteristics between genotypes (Table 1).

The distributions of allele frequencies were 0.163 and 0.386 for CYP2D6*4 and SULT1A1*2, respectively. Since the CYP2D6*4 homozygotes were few, patients with at least one CYP2D6*4 allele were combined in the statistical analyses. Similarly, patients carrying the SULT1A1*2 allele were grouped together. To investigate whether the genotype had a prognostic value, in terms of distant recurrence-free survival, ER-positive and ER-negative patients homozygous for CYP2D6*1 alleles were compared with carriers of the CYP2D6*4 allele, and the patients homozygous for the SULT1A1*1 allele were compared with carriers of the SULT1A1*2 allele. No statistical differences in distant recurrences were found according to genotype (data not shown). To assess the benefit from tamoxifen treatment, distant recurrence-free survival was only calculated in ER-positive patients.

**Figure 1:**
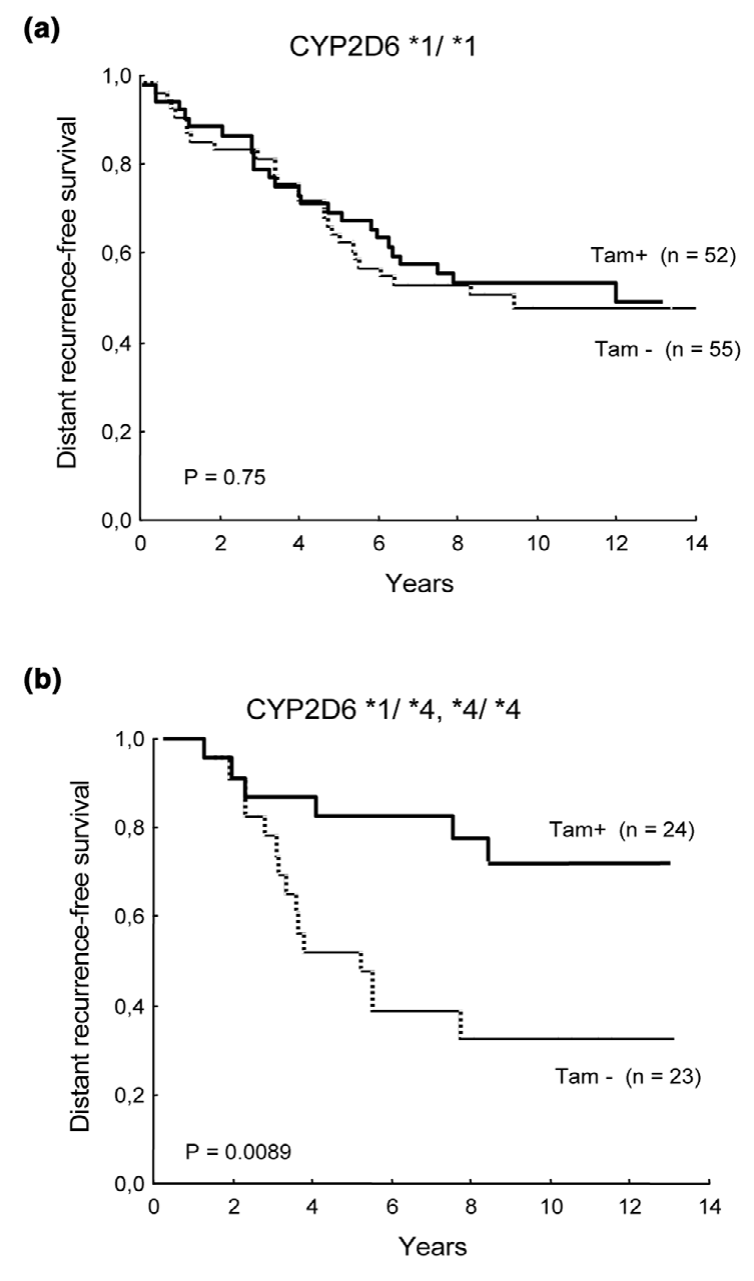
Distant recurrence-free survival among postmenopausal women with oestrogen-receptor-positive breast cancers, in relation to the *CYP2D6 *genotype and adjuvant tamoxifen treatment. Solid line, patients receiving tamoxifen (Tam+); dashed line, patients who did not receive tamoxifen (Tam-). **(a) **Patients homozygous for the CYP2D6*1 allele. The number of events for Tam+ and Tam- were 25 and 27, respectively. **(b)**. Patients homozygous or heterozygous for the CYP2D6*4 allele (null allele). The number of events for Tam+ and Tam- were 6 and 15, respectively.

**Figure 2:**
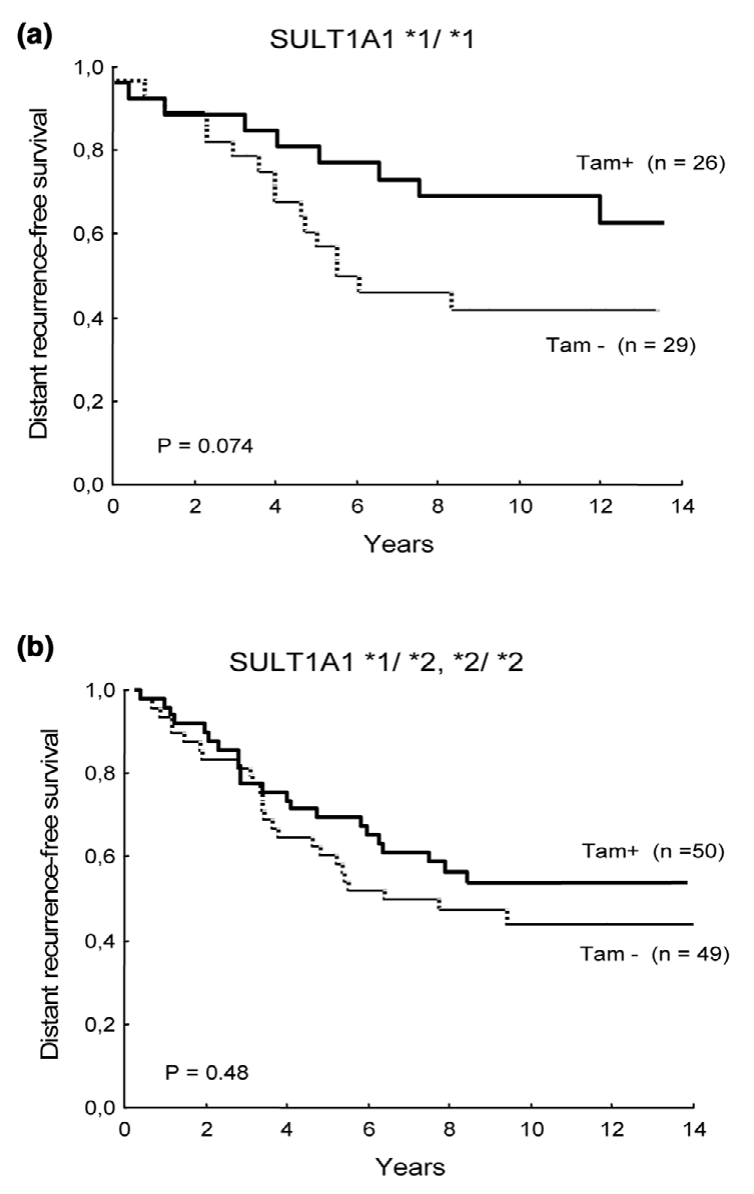
Distant recurrence-free survival of postmenopausal, oestrogen-receptor-positive breast cancer patients in relation to the *SULT1A1 *genotype and adjuvant tamoxifen therapy. Solid line, patients receiving tamoxifen (Tam+); dashed line, patients who did not receive tamoxifen (Tam-). **(a) **Patients homozygous for the SULT1A1*1 allele. The number of events for Tam+ and Tam- were 9 and 16, respectively. **(b) **Patients homozygous or heterozygous for the SULT1A1*2 allele (low-activity allele). The number of events for Tam+ and Tam- were 24 and 26, respectively.

Distant recurrence-free survival for CYP2D6*1 homozygotes, for CYP2D6*4 heterozygotes and homozygotes, for SULT1A1*1 homozygotes, and for SULT1A1*2 heterozygotes and homozygotes are shown in Figs 1a,b and 2a,b, respectively, and are presented in Table 2. Patients possessing at least one CYP2D6*4 allele had better survival when randomised to tamoxifen compared with those who were not randomised to tamoxifen (*P *= 0.0089), as also demonstrated by the significantly decreased relative risk (RR = 0.28, 95% confidence interval [CI] = 0.11–0.74). Among patients homozygous for the CYP2D6*1 genotype, the outcome was approximately equal between tamoxifen-treated and non-tamoxifen-treated patients (*P *= 0.75). A tendency towards improved distant recurrence-free survival in SULT1A1*1 homozygous patients treated with tamoxifen, compared with those receiving no tamoxifen, was found (*P *= 0.074, RR = 0.48, 95% CI = 0.21–1.12) (Fig. 2a). Finally, no influence of tamoxifen therapy on distant recurrence-free survival was found in carriers of the SULT1A1*2 allele (*P *= 0.48) (Fig. 2b).

**Figure 3:**
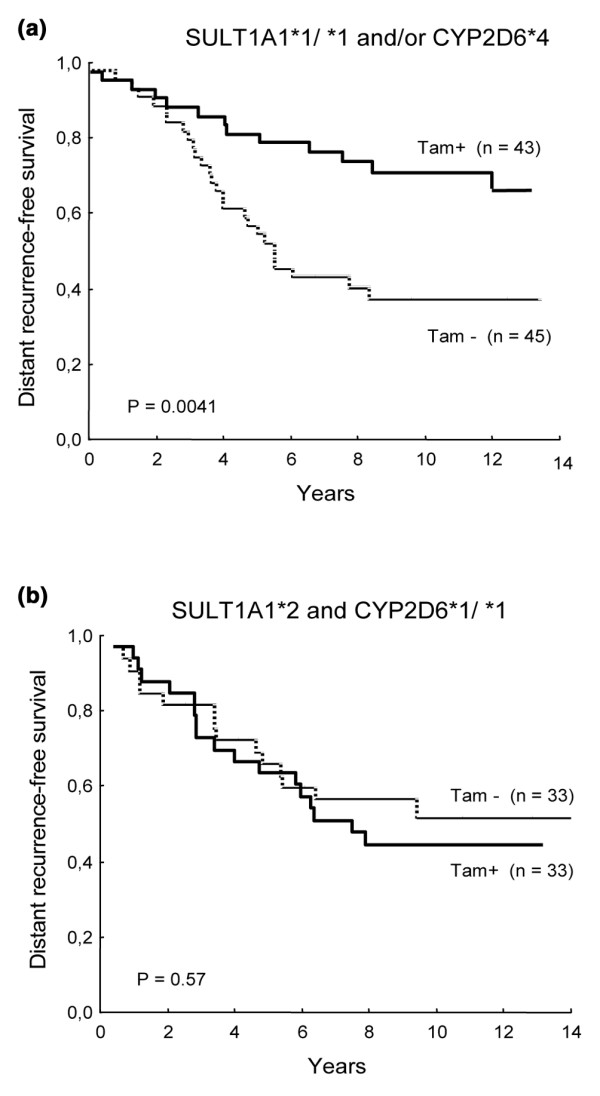
Distant recurrence-free survival of postmenopausal, oestrogen-receptor-positive breast cancer patients with genotypes linked to the benefit from adjuvant tamoxifen therapy. Solid line, patients treated with tamoxifen (Tam+); dashed line, patients not receiving adjuvant tamoxifen therapy (Tam-). **(a) **Patients homozygous for the SULT1A1*1 allele and/or homozygous or heterozygous for the CYP2D6*4 allele. The number of events for Tam+ and Tam- were 15 and 27, respectively. **(b) **Patients homozygous for the CYP2D6*1 allele and homozygous or heterozygous for the SULT1A1*2 allele. The number of events for Tam+ and Tam- were 15 and 18, respectively. The relative risk for distant recurrence was calculated for each genotype; when compared, a significant decrease in relative risk was found for the beneficial genotypes (*P *= 0.018).

The genotypes linked to the benefit from tamoxifen treatment are combined in Fig. 3 as well as in Table 2. In patients harbouring the combination with at least one CYP2D6*4 allele and/or a homozygous SULT1A1*1, tamoxifen treatment significantly improved survival (*P *= 0.0041, RR = 0.38, 95% CI = 0.19–0.74). We also compared non-beneficial alleles (i.e. CYP2D6*1 homozygotes and SULT1A1*2 carriers), and no statistical difference was found in distant recurrence-free survival (*P *= 0.57, RR = 1.22, 95% CI = 0.61–2.4). A comparison of the RRs of distant recurrence, calculated for each combined genotype and adjusted for age, tumour size and lymph node status, demonstrated that the risk reduction with tamoxifen was significantly higher in patients harbouring the combination of CYP2D6*4 and/or SULT1A1*1/*1 (*P *= 0.018) (Fig. 3a,b and Table 2).

## Discussion

We observed a significantly improved benefit from tamoxifen in patients carrying the CYP2D6*4 allele and/or patients homozygous for SULT1A1*1 (*P *= 0.018), compared with patients homozygous for the CYP2D6*1 and carriers of the SULT1A1*2 allele (Fig. 3). To our knowledge this is the first report of the influence of the *CYP2D6 *genotype on tamoxifen therapy, while the influence of the SULT1A1*1 allele has been investigated by Nowell and colleagues [13]. In agreement with the tendency found in the present report, Nowell and colleagues showed that the high-activity allele SULT1A1*1 contributed significantly to tamoxifen response [13]. Those authors suggested that sulfation may affect bioavailability of 4-OH-tamoxifen by reduced clearance of the sulfated metabolite. This may provide a genotype-dependent reservoir of inactivated metabolite, which can be desulfated by steroid sulfatase expressed in breast tumours and can be recovered to the active 4-OH-tamoxifen, leading to a prolonged anti-oestrogen effect [18].

Coller and colleagues [19] and other workers [8,9] have demonstrated in experimental studies that the *CYP2D6 *genotype is a determinant of the ability to form 4-OH-tamoxifen. However, a clinical study by Stearns and colleagues [20] revealed that inhibition of CYP2D6 had no significant effect on 4-OH-tamoxifen concentration. We propose in the present study that genotypes of CYP2D6, which produce a large amount of the ER-active 4-OH-tamoxifen, would be beneficial for the tamoxifen-treated patients. As shown in the present study, patients with at least one CYP2D6*4 allele demonstrated better response to tamoxifen treatment than patients homozygous for the CYP2D6*1 allele. This is in contrast to the main hypotheses where the CYP2D6*1 homozygous patients are supposed to generate the active metabolite 4-OH-tamoxifen more readily and thereby have improved response of tamoxifen. Our results were obtained from a small number of patients, and therefore the association of the genotype and the benefit of tamoxifen treatment may be a coincidence. An absent or decreased CYP2D6-dependent 4-hydroxylation is, however, compensated by CYP2C9 and CYP3A4 to the overall formation of 4-OH-tamoxifen, but the reaction proceeds at a lower rate [19,21].

Interestingly, an additional active tamoxifen metabolite, 4-OH-*N*-desmethyl-tamoxifen (endoxifen), has been recently discovered by Stearns and colleagues [20]. Endoxifen may have clinical relevance since the metabolite inhibits MCF7 cell proliferation with equal potency as does 4-OH-tamoxifen, and it is present in higher plasma concentration in humans than 4-OH-tamoxifen. Endoxifen is mainly synthesised by CYP3A4-mediated *N*-demethylation of tamoxifen and a subsequent 4-hydroxylation by CYP2D6. In humans there are a large number of different polymorphic sites in the *CYP2D6 *gene, and the vast majority is present in a very low frequency.

In the present study we screened for the most common inactivating polymorphism in *CYP2D6*, the CYP2D6*4 allele, which is present at a frequency of approximately 21–29%. Other less common inactive alleles are CYP2D6*3 and CYP2D6*5, representing around 1% and 4%, respectively, of all *CYP2D6 *alleles [22]. Among alleles with decreased enzyme activity the CYP2D6*41 allele identifies a large proportion of the intermediate metabolisers [23]. The restriction fragment length polymorphism technique that we used identifies a restriction site not found in the CYP2D6*4 allele but that is present in other *CYP2D6 *alleles. This results in misclassification of the carriers of CYP2D6*3 and CYP2D6*5 alleles, which could occur in a few cases but would have a minor influence on the results. The definition of CYP2D6*1 used in the present study mainly constitutes the normal activity alleles CYP2D6*1 and CYP2D6*2, which represent a rather high frequency in a Caucasian population [22]. In the regression analysis we combined CYP2D6*4 heterozygotes and homozygotes in one group since the number of homozygous CYP2D6*4 patients was low. Some studies have shown that the hydroxylation ratios are significantly different between the homozygous and heterozygous genotypes, demonstrating intermediate hydroxylation ratios in heterozygous genotypes. There is also support, however, for the hypothesis that only CYP2D6*4 homozygotes will demonstrate altered pharmacokinetics for a given drug [24].

## Conclusion

The variability in distant recurrence-free survival found in endocrine-treated patients may be a result of differences in drug metabolism. The genotype of metabolic enzymes might thus be useful as a guide for adjuvant endocrine treatment of postmenopausal breast cancer patients. However, our results contradict the main hypotheses and the present sample size is relatively small. Our findings therefore need confirmation in a larger cohort.

## Abbreviations

BSA = bovine serum albumin; CI = confidence interval; CYP2D6 = cytochrome P450 2D6; ER = oestrogen receptor; PCR = polymerase chain reaction; RR = relative risk; SULT1A1 = sulfotransferase 1A1.

## Competing interests

The author(s) declare that they have no competing interests.

## Authors' contribution

PW carried out part of the laboratory work and drafted the manuscript. LV carried out part of the laboratory work. OS contributed with the coordination of tumour material and performed the statistical analyses. BN initiated the randomised clinical trial. LS and L-ER provided tumour material and clinical data. SW conceived the study and participated in its design and coordination. All authors read and approved the final version of the manuscript.

## Figures and Tables

**Table 1:** Genotype, tumour characteristics and endocrine therapy of the total study population (*n *= 226), including both oestrogen receptor (ER)-positive and ER-negative patients

Characteristic	Genotype [*n *(%)]					
	
	CYP2D6*1/*1	CYP2D6*1/*4	CYP2D6*4/*4	SULT1A1*1/*1	SULT1A1*1/*2	SULT1A1*2/*2
Nodal involvement^a^, tumour size						
Node-, >30 mm	19 (76.0)	5 (20.0)	1 (4.0)	6 (24.0)	11 (44.0)	8 (32.0)
Node+, ≤ 20 mm	66 (74.2)	18 (20.2)	5 (5.6)	30 (33.7)	49 (55.1)	10 (11.2)
Node+, >20 mm	77 (68.8)	32 (28.6)	3 (2.7)	43 (38.4)	59 (52.7)	10 (8.9)
Receptor status^b^						
ER-negative	50 (74.6)	12 (17.9)	5 (7.5)	23 (34.3)	35 (52.2)	9 (13.4)
ER-positive	109 (69.9)	43 (27.6)	4 (2.6)	56 (35.9)	82 (52.6)	18 (11.5)
Endocrine therapy						
Tamoxifen	77 (68.8)	28 (25.0)	7 (6.3)	37 (33.0)	60 (53.6)	15 (13.4)
No tamoxifen	85 (74.6)	27 (23.7)	2 (1.8)	42 (36.8)	59 (51.8)	13 (11.4)

^a^Nodal involvement: node+, node-positive; node-, node-negative.

^b^ER data from three patients were missing.

**Table 2:** Statistics of oestrogen-receptor-positive patients: association between tamoxifen therapy/no tamoxifen therapy (Tam+/Tam-) and distant recurrence rate, stratified according to genotype

Genotype	Tamoxifen therapy	Number of patients^a^	Number of recurrences	Recurrence rate ratio (95% confidence interval)	*P *value
SULT1A1*1/*1^†^	Tam-	29	16	1.0	
	Tam+	26	9	0.48 (0.21 – 1.12)	0.074
SULT1A1*2^†^	Tam-	49	26	1.0	
	Tam+	50	24	0.82 (0.47 – 1.43)	0.48
CYP2D6*1/*1^‡^	Tam-	55	27	1.0	
	Tam+	52	25	0.91 (0.53– 1.57)	0.75
CYP2D6*4^‡^	Tam-	23	15	1.0	
	Tam+	24	6	0.28 (0.11 – 0.74)	0.0089
SULT1A1*1/*1 and/or CYP2D6*4^§^	Tam-	45	27	1.0	
	Tam+	43	15	0.38 (0.19– 0.74)	0.0041
SULT1A1*2 and CYP2D6*1/*1^§^	Tam-	33	18	1.0	
	Tam+	33	15	1.22 (0.61– 2.40)	0.57

The relative risks of distant recurrence, calculated for each combined genotype are adjusted for age, tumour size and lymph node status.

^a^Follow-up data of two patients were missing.

^†,‡,§^The risk ratio was first calculated separately for each genotype and genotype combination. Second, the test for interaction between the risk ratios was performed by Cox regression: ^†^*P *= 0.27, ^‡^*P *= 0.064, and ^§^*P *= 0.018. The risk ratio for patients not receiving tamoxifen (Tam-) is calculated as 1.0.
